# An assessment of the levels of phthalate esters and metals in the Muledane open dump, Thohoyandou, Limpopo Province, South Africa

**DOI:** 10.1186/1752-153X-2-9

**Published:** 2008-05-12

**Authors:** Adeleke Adeniyi, Matthew Dayomi, Pitso Siebe, Olumuyiwa Okedeyi

**Affiliations:** 1 Department of Chemistry, Lagos State University, Ojo, PMB, 0001, LASU Post Office, Badagry Expressway, Ojo, Lagos, Nigeria; 2 Department of Urban and Regional Planning, School of Environmental Sciences, University of Venda for Science and Technology, Thohoyandou, 0950, Limpopo Province, South Africa

## Abstract

**Background:**

This work reports the determination of the levels of phthalate esters (dimethyl phthalate (DMP), diethyl phthalate (DEP), dibutyl phthalate (DBP), diethyl hexyl phthalate (DEHP)) and metals (lead, cadmium, manganese, zinc, iron, calcium) in composite soil samples. The soil samples were collected randomly within the Muledane open dump, Thohoyandou, Limpopo province, South Africa. Control samples were collected about 200 m away from the open dump. The phthalate esters were separated and determined by capillary gas chromatography with a flame ionization detector, whilst the metals were determined by atomic absorption spectrophotometry.

**Results:**

Open dump values for the phthalate esters and metals to be generally higher in comparison to control samples for DMP, DEP, DBP and DEHP – the mean values calculated were 0.31 ± 0.12, 0.21 ± 0.05, 0.30 ± 0.07, and 0.03 ± 0.01 mg/kg, respectively, for the open dump soil samples. Nonetheless, the mean open dump values for lead, cadmium, manganese, zinc, iron and calcium were 0.07 ± 0.04, 0.003 ± 0.001, 5.02 ± 1.92, 0.31 ± 0.02, 11.62 ± 9.48 and 0.12 ± 0.13 mg/kg, respectively. The results were compared statistically.

**Conclusion:**

Our results revealed that the discarding of wastes into the open dump is a potential source of soil contamination in the immediate vicinity and beyond, *via *dispersal. Increased levels of phthalate esters and metals in the soil pose a risk to public health, plants and animals. Sustained monitoring of these contaminants is recommended, in addition to upgrading the facility to a landfill.

## Background

The dumping of waste on soils has been found to increase their phthalate esters and metals concentrations [1-8]. Soil contaminants may be divided into two groups: organic contaminants, which contain carbon, and inorganic contaminants, which do not contain carbon [9]. The organic contaminants of greatest concern are industrial in origin, and include agricultural pesticides and non-pesticide compounds like phthalate esters; whereas most important inorganic contaminants are metals, originating from industrial processes [2,8,10-13].

The disposal of waste poses major environmental and public health problems in cities across the world [5,14-16]. This has become a source of concern for rural and urban planners [17-21]. With increasing populations, urbanization and spatial growth in South Africa, most open dumps are now close to residential areas. The Muledane open dump in Thohoyandou is now in the heart of Muledane housing estate. Its location presents serious threats to, amongst other things, the quality of ground and surface water *via *contaminant leaching and runoffs [4,5,12,14,22]. It has been documented that organisms inhabiting contaminated soils take up pollutants, which can bioaccumulate in the complex food chain [9,22-24]. Despite this, open dumps remain the preferred management method for the disposal of municipal solid waste in many developing countries [19,20]. Lack of space for dumping solid waste has become a problem for many urban managers, who are concerned about the increasing costs of waste disposal and the possible hazards to water supply and air quality [10,25].

The dialkyl or alkyl aryl esters of 1, 2-benzenedicarboxylic acid – commonly called phthalate esters – have a myriad of commercial uses, and are considered ubiquitous environmental contaminants. Globally, over 8 billion tonnes of phthalate esters are used each year primarily as additives to poly (vinyl) chloride (PVC) plastics, industrial solvents and components of many consumer products [26]. Phthalates have been implicated as possible carcinogenic and teratogenic agents for humans [27-30]. However, it is the possible action of phthalates as endocrine disrupters in humans that has caused the most serious concern [27,31-33]. The toxic effects to humans and wildlife of metals such as cadmium and lead are well known. Even in small amounts humans do not require this category of metals [34]. Similarly, elevated levels of essential metals like zinc, manganese and iron have been found to be toxic [10,35]. Calcium, on the other hand, enhances the toxicity and bioavailability of metals like cadmium and zinc [36,37].

The objective of this study was to evaluate the levels of some phthalate esters (dimethyl phthalate, (DMP), diethyl phthalate, (DEP), dibutyl phthalate (DBP), diethyl hexyl phthalate (DEHP)) and metals (lead, cadmium, manganese, zinc, iron, calcium) in the Muledane open dump soils in relation to control soil samples. This is expected to provide baseline data that will assist the municipal and provincial authorities in adopting and enforcing a sustainable waste disposal and management system especially in South Africa's emerging urban settlements.

## Results and Discussion

The mean soil pH values are 6.55 ± 0.44 and 6.10 ± 0.72 (Table 1) for the open dump and control samples respectively. These values fall within the range of 6.00–7.00 expected for most tropical soils [11,38]. The open dump soil's mean pH value of 6.55 ± 0.44 is however lower than the values 7.40 – 10.4 previously reported for some Lagos dumpsites [1,7]. Soil pH is a very important factor in controlling the mobility and availability of metals. High pH reduces the plant availability of most metals [39]. With a t_cal _value of 1.18 compared to the t_tab _value of 2.31, the soil pH values are non-significantly different at 95% confidence level (Table 1).

**Table 1:** Some characteristics of the sampled soil samples

	**Dump**	**Control**	**t**_cal_
pH	6.55 ± 0.44	6.10 ± 0.72	1.18^ns^
Moisture content (%)	10.89 ± 2.83	9.29 ± 1.51	1.11^ns^
Soil texture	LC	SLC	-

Notes: LC = Loamy clay; SLC = Sand loamy clay; ± SD = standard deviation, t_tab_= 2.31 at p < 0.05; ns = non-significant differences at p < 0.05

The mean percentage moisture content shown in Table 1 reveals values ranging from 9.29 ± 1.51 to 10.89 ± 2.83. These relatively low values are expected of tropical soil sampled before the onset of heavy tropical rains [11]. However, the open dump and control soil samples exhibit non-significant differences at 95% confidence level, an indication of some degree of similarity in soil texture.

The phthalate esters were eluted from the gas chromatographic column in the order: DMP, DEP, DBP, and DEHP. The following retention times (min) were obtained: DMP, 2.25 ± 002; DEP, 3.03 ± 0.03; DBP, 5.73 ± 0.03; DEHP, 0.98 ± 0.05. The method detection limit (MDL) and instrument detection limit (IDL) are as follows (ng/kg): DMP, 40.17 and 20.46; DEP, 48.89 and 20.46; DBP, 87.52 and 3.80; DEHP, 14.61 and 1.09 respectively for MDL and IDL. The percentage recoveries from spiking experiments are: DMP, 89.08 ± 0.51; DEP, 89.95 ± 0.34; DBP, 88.72 ± 0.55; DEHP, 89.04 ± 0.48. The unrestricted dumping of domestic and medical waste materials made of plastic is the likely source of phthalate esters in the soil samples [5,8,20].

Table 2 provides a list of phthalate esters separated and detected in the soil samples as well as the phthalate pollution index (PPI). Similarly, the t-test, statistical analyses of phthalate esters concentration in the open dump and control soil samples are shown in Table 3. The DMP, DEP, DBP and DEHP levels (mg/kg) in the open dump samples are 0.31 ± 0.12, 0.21 ± 0.05, 0.30 ± 0.07 and 0.031 ± 0.01, respectively. However, the control soil samples have values (mg/kg) of 0.20 ± 0.12, 0.18 ± 0.02, 0.23 ± 0.05 and 0.02 ± 0.01 respectively for DMP, DEP, DBP and DEHP.

**Table 2:** Phthalate esters concentrations (mg/Kg) and phthalate pollution index (PPI) in the open dump and control samples

**Phthalate esters**					
**Sampling points**	**DMP**	**DEP**	**DBP**	**DEHP**	**PPI**

1	0.46 (0.09)	0.21 (0.17)	0.35 (0.19)	0.05 (0.02)	0.20 (0.09)
2	0.27 (0.19)	0.16 (0.17)	0.22 (0.30)	0.02 (0.03)	0.12 (0.13)
3	0.29 (0.20)	0.16 (0.16)	0.23 (0.20)	0.02 (0.02)	0.12 (0.11)
4	0.37 (0.40)	0.28 (0.20)	0.39 (0.21)	0.04 (0.04)	0.20 (0.14)
5	0.14 (0.11)	0.22 (0.21)	0.30 (0.27)	0.03 (0.03)	0.13 (0.12)
Mean ± SD	0.31 ± 0.12 (0.20 ± 0.12)	0.21 ± 0.05 (0.18 ± 0.02)	0.30 ± 0.07 (0.23 ± 0.05)	0.03 ± 0.01 (0.02 ± 0.01)	

Notes: DMP = dimethyl phthalate; DEP = diethyl phthalate; DBP = dibutyl phthalate; DEHP = di (ethyl hexyl) phthalate; ND = not detected; Values in parentheses are for the control samples.

**Table 3:** t-test statistical analysis of phthalate esters concentration in the open dump and control soil samples

**DMP**	**DEP**	**DBP**	**DEHP**
0.40^ns^	1.23^ns^	1.04^ns^	0.93^ns^

t_tab_= 2.31 at p < 0.05; ns = non-significant differences at p < 0.05

Biosolids from five treatment plants in greater Vancouver, British Columbia, reveal DMP, DEP, DBP and DEHP values (mg/kg) of 0.13 ± 0.34, 0.15 ± 0.14, 0.15 ± 0.15 and 2.70 ± 2.70 respectively [3]. These values – except for DEHP – are found to be lower than the levels reported for the Muledane open dump soil samples (Table 2). Nevertheless, contaminated soils in Ontario, Canada, as reported [9] have DMP, DEP and DBP levels of < 15 mg/kg, and DEHP burden of < 250 mg/kg. A statistical comparison of DMP, DEP, DBP and DEHP concentrations in the respective soil samples reveal non-significant differences at p < 0.05. A similar trend has been observed before, and may be taken as an indication of low levels of organic contaminant pollution in the open dump [3,8]. Some degree of plastic recycling activities in the vicinity of the open dump may be responsible for this. Of concern, however, is the detection of DEHP in the soil samples, ranging from 0.02 to 0.05 mg/kg in the open dump soils, and 0.02 to 0.03 mg/kg in the control soil samples. DEHP is regarded as having the most deleterious endocrine disrupting capability, resulting in the European Union's (EU) having restricted its use [40].

The maximum admissible concentration (MAC) for the other low molecular weight phthalates, such as DMP, DEP and DBP, has, however, been established by most countries [30,32]. The phthalate pollution index (PPI) for the soil samples is relatively low at 0.12 – 0.20 (open dump) and 0.09 – 0.14 (control). It is instructive to note, however, that the PPI is higher for the open dump soil samples. Although non-significant levels are recorded between open dump and control samples, regular monitoring is imperative, due to the expected increase in urbanization and industrialization of Thohoyandou in the years ahead.

The metal recovery studies to assure quality gave the following the percentage recoveries: Pb, 90.21 ± 0.004; Cd, 95.40 ± 0.005; Mn, 89.94 ± 0.003, Zn, 93.14 ± 0.002, Fe, 91.64 ± 0.004; Ca, 94.71 ± 0.003. The burden of metals (Table 4) in the soil samples collected from the open dump is generally higher than those of samples collected from the control site. This trend has been observed before in open dump soils [1,5,8].

**Table 4:** Metal concentrations (mg/kg), dry mass and metal pollution index (MPI) in the open dump and control soil samples

**Element**							
**Sampling Points**	**Pb**	**Cd**	**Mn**	**Zn**	**Fe**	**Ca**	**MPI**

1	0.03 (0.04)	0.003 (0.004)	7.56 (3.02)	0.27 (0.19)	0.77 (9.38)	0.04 (0.09)	0.13 (0.21)
2	0.03 (0.02)	0.002 (0.002)	3.70 (0.71)	0.19 (0.06)	2.12 (5.74)	0.04 (5.74)	0.12 (0.08)
3	0.12 (0.04)	0.003 (0.004)	2.61 (9.22)	0.29 (0.08)	15.43 (19.04)	0.05 (0.02)	0.24 (0.19)
4	0.05 (0.06)	0.004 (0.005)	5.41 (4.87)	0.28 (0.13)	20.39 (17.52)	0.17 (0.03)	0.32 (0.21)
5	0.10 (0.04)	0.004 (0.003)	5.81 (1.89)	0.51 (0.07)	19.39 (6.75)	0.34 (0.05)	0.45 (0.13)
Mean ± SD	0.07 ± 0.04 (0.04 ± 0.01)	0.003 ± 0.001 (0.004 ± 0.001)	5.02 ± 1.92 (3.94 ± 3.33)	0.31 ± 0.12 (0.11 ± 0.05)	11.62 ± 9.48 (11.69 ± 6.19)	0.12 ± 0.13 (0.04 ± 0.03)	

**Notes**: SD = standard deviation; values in parentheses are for the control soil sample

The mean lead, cadmium, manganese, zinc, iron and calcium levels for the control site are (in mg/kg): 0.04 ± 0.01, 0.004 ± 0.001, 3.94 ± 3.33, 0.11 ± 0.05, 11.69 ± 6.19 and 0.04 ± 0.03 respectively. Whereas the values for the open dump samples are (mg/kg): 0.07 ± 0.04, 0.003 ± 0.001, 5.02 ± 1.92, 0.31 ± 0.12, 11.62 ± 9.48 and 0.21 ± 0.13 for lead, cadmium, manganese, zinc, iron and calcium respectively. There exist statistically significant differences (95% confidence level) in the concentrations of zinc in the soil samples (Table 5). This may be taken as an indication of zinc pollution in the open dump. Waste materials in the open dump containing metals are likely sources of the additional burden when compared to the control levels [8,11,22,28]. The metal concentration levels obtained here were generally lower than the average world values reported earlier [1,8,14,23]. This may not be unconnected with the fact that the Thulamela municipality is still evolving economically.

**Table 5:** t-test statistical analysis of metal concentration in the open dump and control soil samples.

**Pb**	**Cd**	**Mn**	**Zn**	**Fe**	**Ca**
1.63^ns^	1.58^ns^	0.98^ns^	3.44*	0.45^ns^	1.34^ns^

t_tab _= 2.31 at p < 0.05; * = significant differences at p < 0.05; ns = non-significant differences at p < 0.05

The concerns regarding zinc are its toxicity to aquatic species, its ability to bioaccumulate, and the interrelationship of zinc with cadmium and lead [41-43]. Similarly, calcium, iron and zinc play major roles in interfering with cadmium adsorption in the human intestine [44]. Soil losses of soluble calcium salts gradually lead to decalcification of the soil and thus to lower pH. This process may be accelerated under wet conditions [39,45]. Nevertheless, the levels of lead, cadmium, manganese and iron are non-significantly different at 95% confidence level. It is noteworthy that the burden of lead and cadmium in the open dump is relatively high enough to raise public health concerns. These metals are not required even in small amounts by living organisms [33,46]. The metal pollution index (MPI) shown in Table 4 reveals values ranging from 0.13–0.45 for open dump soil samples, and 0.08–21 for the control samples. These values are relatively high and could be a cause of future concern when related to pollution indexes reported earlier in literature [47,48].

The paucity of data on the levels of organic and inorganic pollutants and limit values in the country necessitates reference to values in other countries and organizations. Consequently, results from this study may constitute baseline data to fill this gap.

## Conclusion

The study showed that the dumping of wastes in the open dump has the potential to pollute the environment, especially *via *dispersal. This is revealed by the statistically non-significant differences in the levels of phthalate esters and metals (except for zinc), in the open dump and control soil samples.

We can conclude, however, that the phthalate esters and metals may not presently constitute a serious pollution problem. Nevertheless, the presence of the phthalates and metals as contaminants at these relatively low levels is of concern from the point of view of bioaccumulation. The increased levels of metals and phthalate esters in the soil pose risks to public health, plants and animals, with serious implications for the development of housing, industrial estates and agriculture.

There is need for the Thulamela municipal government to evolve a sustained pollutants monitoring program. Similarly, the open dump should be upgraded to a landfill to cope with the economic growth in the locality.

### Experimental

#### Samples

**Figure 1:**
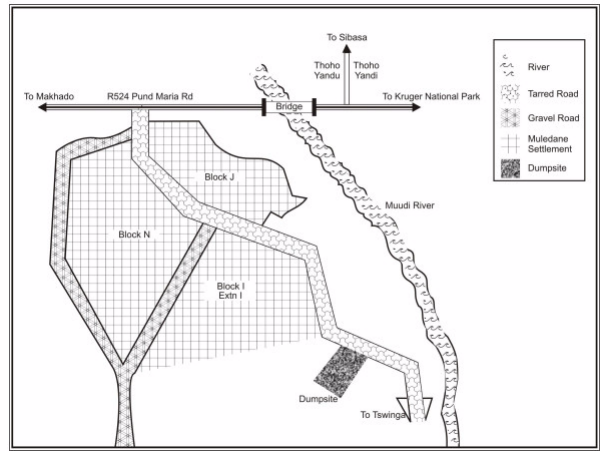
Location of Muledane dumpsite.

Soil samples were collected randomly from the Muledane open dump, located approximately 4.7 km from the central business district (CBD) of Thohoyandou, in the far northern Venda region, of the Limpopo province, South Africa. It is on the Southern side of the Soutpansberg Mountain and falls within the tropical belt of South Africa. Control soil samples were collected from non – dump locations, about 200 m away from the open dump (Figure 1). Ten composite samples each, of control and dump soils were collected randomly within a depth of 15 cm from the soil surface [1,11]. The sampling was carried out between March and May, 2005.

The soil samples collected were air-dried for six days, sieved through 0.45 μm mesh sieve and stored for analysis [7,10,38]. The wastes in the open dump were predominantly domestic, agricultural, and industrial waste materials trucked down to the location. The site started as an uncontrolled open dump in 1989 and was upgraded in 2003 to an appreciably organized open dump by the Thulamela municipality. The open dump is now in the vicinity of the Muledane residential housing estate and poses a potential health threat to the residents.

The choice of the determined potential contaminants – phthalate esters and metals (lead, cadmium, manganese, zinc, iron and calcium) – was informed by the nature of wastes in the site. Calcium was included because it has been implicated in the toxicity and bioavailability of cadmium and zinc [35,36].

### Instrumentation

#### Gas chromatography

The samples were extracted using a soxhlet extractor with 120 ml dichloromethane (DCM). The extracts were left to dry at ambient temperature, reconstituted with 2 ml DCM and cleaned in a short silica gel (Kieselgel 60, 230 – 400 mesh) column, conditioned with hexane and eluted with a benzene/ethyl acetate mixture (95:5), as previously described [49,50].

Separation and determination of the phthalate esters – dimethyl phthalate (DMP), diethyl phthalate (DEP), dibutyl phthalate (DBP) and di-(2-ethyl hexyl) phthalate (DEHP) – were carried out with the Perkin Elmer Clarus 500 gas chromatograph, with a flame ionization detector and capillary column (Col – elite 5–39 m, 0.25 μm – 0.25 mm) (supplied by Perkin Elmer SA (Pty.) Ltd, Cresta, Johannesburg, South Africa).

GC conditions such as oven/inlet temperatures, carrier gas flow, and detector temperature were optimized as follows: oven: initial temperature, 180°C, ramp rate of 12 min, final temperature, 280°C with 2 and 7 min hold times respectively; injector temperature: 180°C, carrier gas set point of 2.0 ml/min. These conditions gave an analysis time of 17.33 min. The stock solution (1000 mg/L) for each ester was prepared in a 20 ml volumetric flask and diluted as appropriate, using a mixture of the phthalates and internal standard (I.S) at 1000 mg/L concentrations with ten replicate injections (1 μL). The response factors were calculated as: Area of the component/Area of I.S

**Figure 2:**
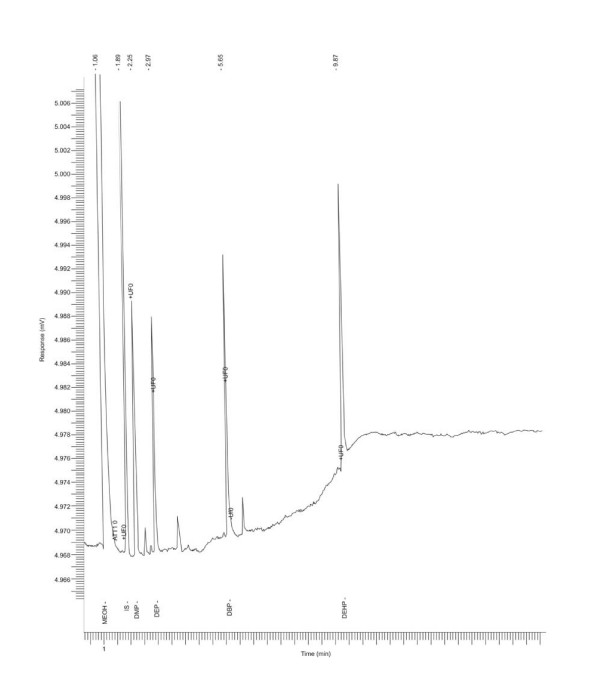
Representative chromatogram of the phthalate esters.

The method detection limit (MDL) was determined following the method described previously [51]:

MDL = Y_b_+3S_b_;

where, Y_b _= blank value; S_b _= standard error of the regression line. The instrument detection limit (IDL) was calculated as described previously [52]. For recovery studies, pre-extracted soil samples were spiked in triplicate with 1 ml, 10 mg/L mixture of the phthalates. A representative chromatogram is shown in Figure 2.

The phthalate pollution index (PPI) of the different sampling events was calculated to compare the total phthalates content in the soil samples according to Usero's *et al*. [53] metal pollution index (MPI) equation:

PPI = (Cf_1 _× C_2_...Cf_n_)^1/n^;

where, Cf_n _= concentration of the phthalates in the samples.

### Atomic absorption spectrometry

The samples were prepared for metal analysis using acid digestion as described earlier [10,11,24,38]. Six metals – lead, cadmium, zinc, manganese, iron and calcium – were determined in an air-acetylene flame with the use of a Varian Spectra AA atomic absorption spectrometer.

The instrument's setting and operational conditions were done in accordance with the manufacturer's specifications, and were calibrated with analytical grade metal standard solutions (1000 mg/L) after appropriate dilutions [10]. 5 g of pre-digested soil samples were spiked with metal standards in triplicate for metal recovery studies as reported earlier [1,10].

To compare the total metal content of the different sampling events the metal pollution index (MPI) was used with the equation:

MPI = (Cf_1_xCf_2 _--- Cf_n_)^1/n^;

where, Cf_n _= concentration of the metal in the sample.

### Physicochemical analysis

Soil moisture content and pH were determined according to the methods enumerated earlier [7].

### Sample preparation for GC

10 g air-dried and sieved (0.45 μm, pore size) soil samples were extracted in triplicate with DCM for 10 hr in a soxhlet extractor. The extract was left to dry on standing at room temperature. A silica gel column was packed in hexane with a top layer (0.5–1.0 ml) of anhydrous sodium sulphate. The phthalates were extracted using 20 ml benzene/ethyl acetate mixture (95:5), and left to dry at room temperature. The dried, clean extracts were reconstituted with 0.5 ml BUBE (I.S). Thereafter 1 μL of each sample was auto-injected into the GC. The response factors obtained from the peak areas were then used to calculate the concentrations of the DMP, DEP, DBP and DEHP contained in the soil samples as described elsewhere [49,52].

### Statistical analysis

Student's t-test was used to study differences in phthalate and metal concentrations between soil samples collected in the open dump and control locations [54].

### Chemicals

Analytical grade reagents phthalates, butyl benzoate (internal standard), metal standard stock solutions (1000 mg/L) and silica gel (Kieselgel, 60, 230–400 mesh) were purchased from Aldrich – Sigma, Merck, Fluka AG and Rochelle chemicals, South Africa.

## Authors' contributions

AA: design, coordination, sampling, bench work, statistical analyses and drafting of manuscript. MD: design, coordination, sampling, statistical analyses and drafting of manuscript. PS: sampling, bench work, statistical analyses and drafting of manuscript. OO: design, drawing of figures, statistical analyses and drafting of manuscript.
